# Weight as an Indication of the Character of the Risk for Life Insurance

**Published:** 1885-05

**Authors:** 


### Editorial.

### Weight as an Indication of the Character of the Risk for Life Insurance.

Dr. Joel Seavems, of Roxbury, in the *Boston Medical and Surgical Journal*, reports his observations upon the influence of weight as an indication of the character of risks for life insurance. The doctor is the medical examiner-in-chief of the Royal Arcanum, a beneficiary organization which has upwards of 50,000 members, and has paid out since January, 1868, over $3,000,000 in life insurance to the families of its deceased members. Any conclusions, therefore, he could present upon the subject of the character of risks would be very instructive

A careful analysis was made of those who, at the time of their admission, were fifteen per cent. or more below the standard weight and then of those who were fifteen per cent. or more *above* that weight. These analyses are presented in tables that are very complete and show, at a glance, the age, height, weight, number of months lived after admission to the order, and the cause of death in each case. Space will not permit us to reproduce these tables, but the doctor's conclusions, briefly stated, are as follows: "For life insurance purposes, men whose weight is above that laid down in the usual tables are better risks than those whose weight is less; that among the latter (light weights) the usual variation of twenty per cent., which is assumed to be within safe limits is not safe, and that if we accept men, especially young men, whose weight is fifteen per cent. below the standard, we are approaching dangerous standing ground, and inviting, as it were, deaths from phthisis and wasting diseases; and when we reflect how great is the mortality from phthisis in all insurance organizations, we cannot too strongly emphasize the necessity of constant vigilance in this direction, and of not only exploring most carefully the chest of such men, but also of taking into account all those other features which I think often precede the changes in lung tissue discernible by the ear, and may be observed at what may be called the pretuberculous stage.

With the heavy weights the case is different. Free from danger of phthisis we must, to be sure, take the greatest care to see that the heart and kidneys are healthy, and that the family history does not point to cerebral disease. With these points well guarded I am satisfied that an excess of twenty-five per cent. in weight is not dangerous in men who have not injured or are not injuring themselves by alcohol."

---

We have received, through the courtesy of Dr. Andrews, the Superintendent, the Fourteenth Annual Report of the Buffalo State Asylum for the Insane, for the year 1884. The Report includes the reports of the Board of Managers, of the

Treasurer, of the Steward, of the Matron, and also of the Superintendent, supplemented with statistical tables showing the admissions, dismissions and the effects of treatment. The pamphlet is unusually full of interesting matter. It shows the excellent system and management of this important institution, which, thus early in its history, has become a pride to the city and to the State.

---

MESSRS. LEA BROTHERS & Co. have issued a handsomely printed octavo volume, which, under the title "One Hundred Years of publishing 1785–1885," gives an interesting history of the great house which, a century ago, was founded in Philadelphia by Matthew Curey. The Lea's came into the firm in 1821. The record of the house has been one of honorable dealing, unflagging industry and excellent judgment in so much that their imprint is in some sort an indication of the worthiness of the volume in which it appears.

---

IT IS with great pleasure that we learn that the *Index Medicus* is to be continued, the enterprising house of George I. Davis, of Detroit, having assumed the responsibility of its publication. Although, in the past, financially unprofitable, it is of genuine value to the profession, giving, as it does, complete reference to what is published in all parts of the world. It should be found in every public library and should number among its subscribers every medical society, if not every progressive practitioner.

---

SEVERAL of the State Boards now refuse to recognize the diplomas of medical colleges which require no matriculation examination. The adoption, by the American Medical Association, of the resolution "that no person who shall hereafter graduate from a medical school where literary education is *not* a prerequisite to such graduation shall be eligible to be a delegate," will be another step in the right direction.

Dr. Davidson has opened, near the corner of Genesee, Huron and Main streets, a "Hospital for Diseases of the Skin." This is intended for the convenience of out-patients. The poor are treated free upon the recommendation of any physician. Those whose condition requires continuous observations are admitted to the Buffalo Hospital of the Sisters of Charity.

---

Dr. Cust. H. Lovelace, of Cashon, Tenn., says: "I have used Papine in my practice for some time and am thoroughly satisfied it is the most desirable preparation of opium I have ever used, and more reliable than any other form of opium. I believe it will speedily displace every other member of the opiate family."

---

Mr. A. L. Arey's lectures upon "Electricity," at the Niagara Medical College, have proved highly interesting and instructive. The experimental illustrations have been very attractive. We understand that the lectures are to be continued every Thursday evening through this month.

---

We would call attention to John Wyeth & Bro.'s advertisement in this number of a Portable Antiseptic Dressing. The tablets make a solution which is exactly correct as to strength, and we find them a great convenience.

